# Effect of stocking density and vitamin E or zinc supplementation on growth, physiology, gene expression, and economic efficiency of growing broiler chicks

**DOI:** 10.1007/s11250-022-03382-6

**Published:** 2022-11-26

**Authors:** Seham F. Shehata, Samar H. Baloza, Mohamed M. M. Elsokary, Nesrein M. Hashem, Maha M. Khawanda

**Affiliations:** 1 Veterinary Economics and Farm Management, Department of Animal Wealth Development, Faculty of Veterinary Medicine, Benha University, Benha, PO 137386 Egypt; 2 Genetics and Genetic Engineering, Department of Animal Wealth Development, Faculty of Veterinary Medicine, Benha University, Benha, PO 137386 Egypt; 3 Department of Theriogenology, Faculty of Veterinary Medicine, Benha University, Benha, PO 137386 Egypt; 4 Veterinary Medicine & Food Security Research Group, Faculty of Health Sciences, Higher Colleges of Technology, 17155 Abu Dhabi, United Arab Emirates; 5 Department of Animal and Fish Production, Faculty of Agriculture, Alexandria University, Alexandria, 21545 Egypt; 6 Physiology Department, Faculty of Veterinary Medicine, Benha University, Benha, PO 137386 Egypt

**Keywords:** Broiler, Stocking density stress, Zinc, Vitamin E, Growth, Profit

## Abstract

A total of 636 1-day-old male Cobb chicks were randomly assigned to seven treatments. The chicks were offered feed and water ad libitum throughout the experimental period. The first three groups included different stocking densities of broiler birds (low stocking density, LSD: 23 kg/m^2^; medium stocking density, MSD: 34 kg birds/m^2^; and high stocking density HSD: 39 kg birds/m^2^). The LSD group was considered a control group. The other four groups included MSD or HSD broiler birds supplemented with either Vit E (100 mg/kg DM diet; MSDVE and HSDVE) or Zn (100 mg/kg DM diet; MSDZn and HSDZn) in their basal diet. The main findings indicated that HSD and MSD negatively affected (*p* < 0.05) all variables under investigation compared with LSD. Compared with LSD, broiler birds in the MSD and HSD groups had lower body weights and higher feed conversion ratios, higher concentrations of blood plasma hormones (triiodothyronine thyroxine and corticosterone), and downregulated expression levels of hepatic growth hormone and insulin-like growth factor-l. In addition, broiler birds stocked at medium or high densities resulted in less economic return and profit. Vit E or Zn supplementation to broiler birds stocked at medium or high densities significantly reversed all adverse effects of HSD (> 23 kg/m^2^) on growth performance, hormones, and gene expression. It could be recommended that adding Zn at a level of 100 mg/kg per DM diet allows increasing the stocking density of broiler birds from 23 kg/m^2^ to 34 birds/m^2^ while maintaining the birds, welfare and economic profit.

## Introduction

Worldwide poultry production has a significant economic role and remains at the top of meat production industries. Recently, poultry production has rapidly become more intensive to support the resilience of global food supply chains and meet the increased demand for animal protein sources. Thus, in the modern poultry industry, broilers are usually reared at a high stocking density (HSD) to maximize the number of chickens produced per square meter of space to decrease the cost of production and to achieve sufficient economic returns (Abudabos et al. 2013; Goo et al. 2019; Li et al. 2019). However, HSD of birds is usually related to negative effects on birds’ welfare and health and thus their productivity, which may negate the outputs and profits of intensive production (Simitzis et al. 2012).

Growth hormone (GH), triiodothyronine (T_3_), thyroxine (T_4_), and insulin-like growth factor-1 (IGF-1) are the main hormones that determine the expected growth in chickens (Scanes 2009). GH directly works on growth, development, and metabolism (Kim 2010). GH activates hepatic IGF-I release indirectly by combining GH receptors on the liver membrane. IGF-1 is a multifunctional polypeptide hormone that significantly affects growth and development and is more like the insulin hormone. An alteration in this gene can affect its expression level and thus affect the developmental characteristics of animals. Therefore, IGF-1 can be a better indicator for growth (Duclos 2005). Among trace minerals, specific importance is given to Zn due to its involvement in several important biological events such as maintaining growth performance, boosting the immune system, and supporting the antioxidant defense system (Shah et al. 2018; Hidayat et al. 2020). It regulates the process of skeletal development, membrane protection, prostaglandin metabolism, and lipid metabolism. Zn is involved in the activity of more enzymes, and the metalloenzymes (Tatiane et al. 2020). Of vital vitamins for growing chicks, Vit E is one of the essential vitamins in poultry nutrition, and it is considered a natural antioxidant usually provided in the form of synthetic alpha-tocopherol (Vieira et al. 2021). It is well known that vitamin E supplementation has a protective function in such stressful conditions (Lohakare et al. 2016). Vitamin E is a fat-soluble vitamin known to be a biological membranous lipid component and natural antioxidant. Fundamentally, vitamin E is located in the membrane lipid bilayer hydrocarbon part towards the membrane interface near to oxidase enzymes, which initiate free radicals production (Khalifa et al. 2021). These free radicals are generated due to normal cell activity and increase due to several stress factors. Thus, vitamin E protects cells and tissues from free radicals that cause oxidative damage to the cells (Part et al. 2004).

Supplementing Vit E to feed could overcome the undesirable effects of overcrowding stress and positively impact broiler chickens’ productive performances (Khalifa et al. 2021; Part et al. 2004). More information is required to understand the mechanisms by which HSD can biologically affect productivity and the birds’ health. Also, previous studies have not adequately addressed the economic efficiency of rearing birds at a HSD. In this study, we focused on studying the effects of different levels of stocking densities (SD) on growth and stress-related hormones to identify how these physiological processes are affected by overcrowding. We also investigated whether organic Vit E or Zn supplementation could alleviate the detrimental effects of HSD on the growth performance, physiological parameters, and gene expression of broiler birds. We chose these two supplementations based on their important biological roles in poultry (Shah et al. 2018; Hidayat et al. 2020). Finally, we evaluated the impacts of HSD and the expected benefits of adding Vit E or Zn economically to consider the profitability of one broiler fattening cycle.

## Materials and methods

### Birds and diets

This study was performed at the Center of Experimental Animal Research, Faculty of Veterinary Medicine, Benha University, Egypt, during the period from September 8 to October 20 in 2020. All procedures and the research protocol were carried out following the guidelines of the Local Committee for Experimental Animal Care and were confirmed by the ethics of the Institutional Animals Care and Use committee Research Ethics Board, Faculty of Veterinary Medicine, Benha University, under ethical number BUFVTM 08–06-21.

A total of 636 male, 1-day-old chick Cobb-500 broilers were obtained from local hatcheries. All chicks were subjected to the same managerial and hygienic housing conditions. The chicks were transported, weighed, wing banded, and then housed in well-ventilated litter floor pens and stocked in an area 50 cm high × 100 cm wide × 100 cm long. The chicks were exposed to a near-continuous photoperiod length of 23 L:1D with rotation. During the experimental period (6 weeks), the chicks received a typical feeding program that covered their nutritional requirements during different growth phases. The chicks were fed corn-soybean meal-based-basal diets and formulated to meet the nutritional specifications of the Cobb-500 broilers management guide (avialable at: https://www.cobb-vantress.com/assets/5a88f2e793/Broiler-Performance-Nutrition-Supplement.pdf). The feeding program included a starter diet (offered during the first and second weeks of age), grower diet (offered during the third and fourth weeks of age), and finisher diet (offered during the fifth and sixth weeks of age) in a pelleted form. The ingredients and calculated chemical composition of each basal diet are presented in Table 1.

**Table 1:** Ingredients and calculated chemical composition of the basal diets used during different growth phases of broiler chicks

Items	Starter diet	Grower diet	Finisher diet
Yellow corn	54.67	58.28	62.62
Soybean meal, 46%	36	33.8	28.9
Vegetable oil	2.5	3.5	4.5
Corn gluten meal, 60%	2	0	0
Dicalcium phosphate	1.7	1.45	1.33
Limestone	1.45	1.35	1.2
l-Lysine	0.33	0.29	0.23
Sodium chloride	0.32	0.3	0.3
Vit & min premix^1^	0.3	0.3	0.3
dl-Methionine	0.28	0.27	0.23
Sodium bicarbonate	0.19	0.17	0.17
Anti-coccidian	0.05	0.05	0.05
Anti-mycotoxin	0.05	0.05	0.05
Anti-clostridia	0.03	0.03	0.03
l-Threonine	0.03	0.04	0
Energy enzyme	0.02	0.04	0.01
Lysomax	0.01	0.01	0.01
Phytase enzyme	0.01	0.01	0.01
Protease B	0.01	0.01	0.01
Choline chloride	0.05	0.05	0.05
Calculated composition			
Crude protein %	23.02	21.03	19.03
MEn kcal/kg	3053.85	3152.05	3224.10
Crude fiber %	2.27	2.25	3.13
Lysine %	1.35	1.25	1.09
Methionine %	0.63	0.59	0.54
Methionine + cysteine%	1.02	0.95	0.86
Threonine %	0.94	0.88	0.77
Calcium %	1.05	0.95	0.85
Available phosphorus %	0.50	0.45	0.42

^1^Each 3 kg contained Vit. A 12,000,000 IU, Vit. D3 2,000,000 IU, Vit. E 10,000 mg, Vit. K3 2000 mg, Vit. B 11,000 mg, Vit. B2 5000 mg, Vit. B6 1500 mg, Vit. B12 10 mg, biotin 50 mg, pantothenic acid 10,000 mg, nicotinic acid 30,000 mg, folic acid 1000 mg, manganese 60,000 mg, zinc 50,000 mg, iron 30,000 mg, copper 10,000 mg, iodine 1000 mg, selenium 100 mg, cobalt 100 mg, carrier (CaCo3) added to make the total 3 kg

The chicks were distributed randomly into seven treatment groups, with six replicate pens each, with different SDs and dietary supplementations, as follows: (1) low SD (LSD: 10 chicks/m^2^; control), (2) medium SD (MSD: 15 chicks/m^2^), (3) high SD (HSD: 17 chicks/m^2^), (4) MSD (15 chicks/m^2^) supplemented with 100 mg Vit E/kg DM (MSDVE), (5) HSD (17 chicks/m^2^) supplemented with 100 mg Vit E/kg DM (HSDVE), (6) MSD (15 chicks/m^2^) supplemented with 100 mg Zn/kg DM (MSDZn), and (7) HSD (17 chicks/m^2^) supplemented with a 100 mg Zn/kg DM diet (HSDZn).

Dietary supplementations, Vit E or Zn, were added to all three basal diets at a 100 mg/kg DM diet level. Vit E, alpha-tocopherol acetate, was obtained from Sigma Al-drich Co., USA. Zn mineral and the zinc amino acid complex were obtained from Multi vita Co., Egypt, with a commercial name Availa-Zn 120*.*

### Evaluation of growth performance

Bodyweight (BW) and body weight gain (BWG) were calculated according to Mohammed et al. (2021a), $\mathrm{BWG}=\mathrm{BW}2-\mathrm{BW}1$ Feed intake (FI) was calculated as the difference between the offered feed’s weight and the refused feed’s weight. The feed conversion ratio (FCR) was calculated $\mathrm{FCR}\:=\:\mathrm{FI}\;(\mathrm g/\mathrm{bird}/\mathrm{week})/\mathrm{BWG}\;(\mathrm g/\mathrm{bird}/\mathrm{week})$  according to Shehata et al. (2021).

### Evaluation of economic efficiency

Economic efficiency comprising the costs of production and return parameters was estimated. Costs of production included total costs (TC), which consisted of total variable costs (TVC) and total fixed costs (TFC). TVC involved the cost of feed consumed, veterinary management, labor, price of chicks, water and electricity, and litter (Al-khalaifah et al. 2020). This was estimated as an average value for each bird in each group per LE (1 USD ≈ 15.67 LE) during the experimental period. TFC included depreciation of the building and equipment.

Total feed cost = total FI per single bird × price/kg (Tareen et al. 2017). The return parameters included total return (TR = live BW and net profit (NP = TR-TC)) (CO and KI 2015). Using the previously mentioned cost and return parameters, economic efficiency measures were calculated as follows: benefit cost ratio (BCR) = TR (L.E/chick/group)/TC (L.E/chick/group), TR (L.E/chick/group/TVC (L.E/chick/group), NP/TC, NP/TVC, BW/kg (Final BW/1000), and WG/kg (Final BWG/1000) (Mohammed et al. 2021b).

### Analysis of mRNA expression of growth hormone (GH) and insulin-like growth factor-1 (IGF-1) genes

At the end of the experiment (week 6), two randomly selected birds of each replicate (*n* = 12 birds/treatment) were slaughtered. Liver samples were immediately stabilized in an RNA stabilization reagent (RNA Later solution; 10 μL per 1 mg of hepatic tissue, Qiagen-GmbH, Germany), and stored at − 80 °C for subsequent total RNA extraction. A total RNA purification kit (Easy Red TM, Intron Biotechnology, Korea) was used for total RNA extraction following the manufacturer’s protocol; that is, 100-mg hepatic tissue was placed in a microcentrifuge tube with 750 μL Trizol solution and homogenized with Rotor Tissue Ruptor (Qiagen, GmbH, Germany). The quantity and purity of the RNA were examined by determining the absorbance in a Spectro Star Nanodrop spectrophotometer (BMG Lab Tec, GmbH, Germany). An RNA concentration of 40 μg/ml corresponds to an absorbance value of 1.0 at 260 nm. Pure RNA has an A260/A280 ratio of 1.8–2.0. A 2 × Reverse Transcriptase Master Mix (Applied Biosystems, USA) was used to synthesize cDNA following the manufacturer’s directions. Relative quantification of the m-RNA expression for the studied genes was carried out via real-time PCR with SYBR green. In a 20 μL reaction mixture containing 10 μL SYBR Green qPCR Master Mix (TOPreal™ qPCR 2X PreMIX), 1 μL of 1 μg/μL cDNA, 1 μM of each forward and reverse primer, and nuclease free water were added to reach 20 μL. Reactions were then made on an Applied Biosystem 7500 Fast Real-time under the following conditions: initial heating at 95 °C for 10 min, 40 cycles of 95 °C for 15 s then 60 °C for 1 min. The primer sequences of GH, IGF-1, and β-actin (used as a housekeeping gene) were 5’-AAGGGATCCAAGCTCCTGAT-3’ (5’-3’ sequence forward) and 5’-ATAACCACGTCCCTCAGTGC-3’ (5’-3’ sequence reverse); 5’-CACCTAAATCTGCACGCT-3’ (5’-3’ sequence forward) and 5’-CTTGTGGATGGCATGATCT-3’(5’-3’ sequence reverse) and 5’- ACCCCAAAGCCAACAGA-3’ (5’-3’ sequence forward) and 5’-CCAGAGTCCATCACAATACC -3’ (5’-3’ sequence reverse), respectively (Gasparino et al. 2014). The PCR primers were manufactured by Invitrogen (Thermo Fisher Scientific, USA). Changes in gene expression were measured from the cycle threshold (Ct) values obtained relative to those provided by real-time PCR instrumentation using the 2^−ΔΔCt^ calculation, where ΔCt indicates the changes in Ct in target genes compared with those in a reference (housekeeping) gene (Schmittgen and Livak 2008). The Ct is defined as the time when the measured fluorescence rises above the background fluorescence and serves as a tool for calculating the starting template amount in each sample.

### Blood samples for determination of the plasma level of growth-related hormones

At weeks 3 and 6 of the experimental period, a blood sample was collected from the jugular vein of two randomly selected birds of each replicate (*n* = 12 birds/treatment). Samples were retrieved in vacutainer tubes (coated with heparin) and then centrifuged at 3000 rpm for 15–30 min. Plasma was carefully collected and kept frozen at − 20 °C until subsequent analyses.

Concentrations of plasma glucose were determined using an autoanalyzer detector colorimetric procedure according to the instructions provided by the manufacturer (Biodiagnostic Company Giza, Egypt). Concentrations of blood plasma thyroxin (T_4_), triiodothyronine (T_3_) (Guangzhou Wondfo Biotech Co., Ltd. No.8 Lizhishan Road, Science City, Luogang District, 510,663, Guangzhou, P.R. China), and corticosterone (corticosterone: ADI-900–097 Enzo Life Sciences, Inc., Madison Avenue New York, NY, 10,022, USA) were measured using commercial kits following the provided analysis instructions. The sensitivity of the methods was 0.39 ng/ml, 0.01 μg/ml, and 0.027 ng/ml for T_3_, T_4_, and corticosterone, respectively.

### Statistical analysis

The results were statistically analyzed using IBM SPSS version 22. All data were subjected to a one-way ANOVA to detect the differences among the different treatment groups. Contrast analysis was carried out using the control/LSD, MSD, and HSD experimental groups to identify the effects of different SDs on different independent variables regardless of the effect of supplementation. Furthermore, the contrast analysis was carried out to identify the effects of each supplementation (Vit E or Zn) on different independent variables compared with the LSD group. The differences among the means of the experimental groups were detected using Tukey’s test at a 5% probability level. The obtained results are shown as the mean ± pooled standard error of the mean.

## Results

### Growth performance

Table 2 shows the effects of different SDs and the influence of Vit E or Zn supplementation to MSD or HSD birds on the BW, BWG, FI, and FCR of broiler birds during the experimental period. SDs of broiler birds significantly affected the BW, BWG, FI, and FCR of birds. Birds in the MSD and HSD groups showed significant decreases in all growth parameters than birds in the LDS group. At the grower phase, the addition of either Vit E or Zn to the birds stocked at medium or high density (MSDVE, HSDEV, MSDZn, and HSDZn) resulted in similar BW compared with the LDS (control) group and significantly higher BW compared with the MSD and LSD groups. However, this trend was only observed in the MSDVE and MSDZn groups at the finisher phase (week 6). The highest (*p* < 0.05) overall means of BWG were observed in the LSD, MSDZn, and MSDVE groups. The addition of either Vit E or Zn to the birds stocked at high density (HSDEV and HSDZn) significantly improved the overall means of BWG compared with those stocked at the same densities but without supplementations.

**Table 2:** Effect of different stocking densities (SDs) and the influence of vitamin E (Vit E) or zinc (Zn) supplementation to medium or high stocked density birds on body weight (BW), body weight gain (BWG), feed intake (FI), and the feed conversion ratio (FCR) of broiler birds during the experimental period

Treatment (T)												
Items	SD			SD-treated Vit E		SD-treated Zn		Pooled SEM	*P*-value			
	LSD	MSD	HSD	MSDVE	HSDVE	MSDZn	HSDZn		T^1^	C vs SD	C vs SD + Vit E	C vs SD + Zinc
BW (gm)												
IBW	49.04	49.22	49.27	49.33	49.44	49.59	50.03	0.29	NS	0.99	0.98	0.85
Wk 2. starter phase	489.03	482.67	486.62	489.28	484.51	518.78	508.24	6.13	NS	0.99	1.00	0.61
Wk 4, grower phase	1449.33^a^	1298.67^b^	1285.00^b^	1413.67^ab^	1385.33^ab^	1589.67^a^	1520.67^a^	9.55	*	< 0.01	0.40	0.02
Wk 6, finisher phase	2314.87^a^	2031.92^c^	2017.38^d^	2258.00^ab^	2132.67^bc^	2348.67^a^	2168.00^b^	8.42	**	< 0.01	< 0.01	0.20
BWG (gm)												
Wk 2, starter phase	298.65	291.79	297.42	302.90	290.44	314.44	310.09	5.72	NS	1.00	1.00	0.88
Wk 4, grower phase	547.13^ab^	407.13^c^	400.33^c^	457.69^b^	542.67^ab^	652.82^a^	639.05^a^	9.34	*	< 0.01	0.44	0.03
Wk 6, finisher phase	492.87	328.92	353.38	388.00	339.33	487.67	340.67	17.50	NS	0.08	0.15	0.53
Overall	2265.8^a^	1982.7^c^	1968.1^d^	2208.7^ab^	2083.22^bc^	2299.08^a^	2117.97^b^	8.51	**	< 0.01	< 0.01	0.21
FI (gm)												
Wk 2, starter phase	383.67	367.67	373.00	364.33	373.67	365.00	382.00	3.74	NS	0.70	0.63	0.84
Wk 4, grower phase	670.00^b^	718.33^b^	742.67^ab^	805.33^a^	764.67^ab^	861.00^a^	767.00^ab^	16.26	*	0.67	0.18	0.07
Wk 6, finisher phase	880.00	865.00	885.33	912.67	906.33	960.00	890.00	6.09	NS	0.01	0.47	0.15
Overall	3479.33^b^	3456.0^b^	3562.0^ab^	3671.0^a^	3577.0^ab^	3784.7^a^	3611.3^ab^	22.63	*	0.98	0.24	0.04
FCR												
Wk 2, starter phase	1.29	1.26	1.25	1.20	1.29	1.16	1.26	0.23	NS	0.98	0.96	0.75
Wk 4, grower phase	1.23^ab^	1.78^ab^	1.87^a^	1.77^ab^	1.41^ab^	1.32^ab^	1.20^b^	0.05	*	0.13	0.17	1.00
Wk 6, finisher phase	1.89	2.71	2.58	2.36	2.68	1.98	2.79	0.12	NS	0.24	0.38	0.57
Overall	1.54^c^	1.74^ab^	1.81^a^	1.66^b^	1.72^ab^	1.65^bc^	1.71^ab^	0.01	**	< 0.01	< 0.01	< 0.01

^1^Means within a row with different superscripts (a,b,c) were significantly different (*p < 0.05, **p < 0.01, and *NS*, not significant). *SD*, stocking density; *LSD*, low stocked denisity; *MSD*, medium stocked density; *HSD*, high stocked density; *MSDVE*, medium stocked density birds supplemented with Vit E; *HSDVE*, high stocked density birds supplemented with Vit E; *MSDZn*, medium stocked density birds supplemented with Zn; *HSDZn*, high stocked density birds supplemented with Zn; *IBW*, initial body weight; *FBW*, final body weight; *BWG*, body weight gain; *FI*, feed intake; *FCR*, feed conversion rate; and *SEM*, standard error of the mean. WK2: at the end of the starter phase, WK4: at the end of grower phase, WK6: at the end of finisher phase

Adding either Vit E or Zn to the birds stocked at MSD or HSD (MSDVE, HSDEV, MSDZn, and HSDZn) significantly improved FI compared with the MSD and HSD groups during the finisher phase (weeks 4 to 6 of age). However, the highest overall means of FI were observed in the MSDZn and MSDVE groups. Among the different groups, the lowest (better feed efficiency) overall means of FCR were observed in the LSD, MSDZn, and MSDVE groups.

### Metabolism and gene expression

Table 3 shows the effect of different SDs and the influence of Vit E or Zn supplementation to medium or high stocked birds on blood plasma glucose, T_3_, T_4_, and corticosterone, and liver m-RNA expression of GH and IGF-l genes of broiler birds. The SD of broiler birds had significant effects on the blood plasma glucose, T_3_, T_4_, and corticosterone. At week 3 of age, blood plasma glucose was not affected by the SD; however, significant increases in blood plasma glucose concentrations were observed in the MSD and HSD groups compared with the LSD group. The concentrations of blood plasma T_3_, T_4_, and corticosterone of broiler birds in the MSD and HSD groups increased significantly compared to those recorded for the LSD group either at week three or week 6 of age.

**Table 3:** Effect of different stocking densities (SDs) and the influence of vitamin E (Vit E) or zinc (Zn) supplementation to medium or high stocked birds on blood plasma glucose, stress-related hormones (*T*_*3*_, triiodothyronine; *T*_*4*_, thyroxin; and *cortico*, corticosterone), and liver m-RNA expression (GH: growth hormone, IGF-l: and insulin-like growth factor-l) of broiler birds

Treatment (T)												
Items		SD		SD-treated Vit E		SD-treated Zn		Pooled SEM	*P*-value			
	LSD	MSD	HSD	MSDVE	HSDVE	MSDZn	HSDZn		T^1^	C vs SD	C vs SD + Vit E	C vs SD + zinc
At Wk 3												
Glucose (mg/dl)	217.75^a^	215.0^a^	216.33^a^	207.3^b^	205.0^b^	208.3^b^	204.6^b^	0.58	*	< 0.01	0.83	< 0.01
T_3_ (ng/ml)	47.38^b^	75.05^a^	76.33^a^	42.96^b^	46.5^b^	44.01^b^	47.03^b^	0.16	*	< 0.01	< 0.01	< 0.01
T_4_ (μg/ml)	0.53^b^	0.78^a^	0.779^a^	0.54^b^	0.53^b^	0.55^b^	0.52^ab^	0.004	*	1.00	0.97	0.07
Cortico (ng/ml)	3.10^b^	6.7^a^	8.02^a^	3.10^b^	3.30^b^	3.00^b^	3.10^b^	0.07	*	< 0.01	0.99	0.99
At Wk 6												
Glucose (mg/dl)	159.50^c^	185.33^b^	202.66^a^	153.00^c^	152.66^c^	154.33^c^	156.00^c^	1.06	**	< 0.01	0.06	0.81
T_3_ (ng/ml)	47.11^bc^	79.20^a^	80.48^a^	46.00^b^	54.40^c^	46.00^b^	49.53^bc^	0.21	**	< 0.01	0.65	< 0.01
T_4_ (μg/ml)	0.85^b^	1.20^a^	1.60^a^	0.43^c^	0.85^c^	0.41^c^	0.78^c^	0.01	**	< 0.01	< 0.01	0.80
Corticos (ng/ml)	9.06^b^	13.00^a^	16.06^a^	9.23^b^	8.86^b^	9.76^b^	9.70^b^	0.21	*	< 0.01	1.00	0.78
Liver m-RNA gene expression												
GH	1.19^a^	0.78^b^	0.54^c^	0.88^ab^	0.84^ab^	0.89^ab^	0.77^b^	0.03	*	< 0.01	0.02	0.01
IGF-1	1.04^a^	0.71^b^	0.69^b^	0.93^ab^	0.75^b^	0.88^ab^	0.75^b^	0.02	*	< 0.01	0.02	0.01

^1^Means within a row (a,b,c) are significantly different (*p < 0.05, **p < 0.01, and *NS*, not significant). ^1^Means within a row with different superscripts (a,b,c) significantly differ (*p < 0.05, **p < 0.01, and *NS*, not significant). *SD*, stocking density; *LSD*, low stocked denisity; *MSD*, medium stocked density; *HSD*, high stocked density; *MSDVE*, medium stocked density birds supplemented with Vit E; *HSDVE*, high stocked density birds supplemented with Vit E; MSDZn, medium stocked density birds supplemented with Zn; *HSDZn*, high stocked density birds supplemented with Zn; *Wk*, week; and *SEM*, standard error of the mean

At week 3 of age, blood plasma glucose was significantly decreased in the MSDZn, HSDZn, MSDVE, and HSDVE groups at the third and sixth weeks of age compared with the LSD, MSD, and HSD groups. This trend changed at week 6 of age, as the differences between Vit E- or Zn-supplemented groups and the LSD group disappeared while remaining lower than that observed in the MSD and HSD groups. The concentrations of T_3_ did not differ between the LSD group and all groups supplemented with either Vit E or Zn, whereas the highest values were observed in the MSD and HSD groups either at week 3 or week 6 of age. At week 3 of age, the concentrations of blood plasma T_4_ and corticosterone of all Vit E- or Zn-supplemented groups did not differ from those observed in the LSD group, whereas the highest values were observed in the MSD and HSD groups. At week 6 of age, all Vit E- or Zn-supplemented groups had significantly decreased blood plasma T_4_ and corticosterone concentrations compared with the LSD group, whereas the highest values were observed in the MSD and HSD groups.

For the relative expression levels of mRNAs of GH and IGF-1 genes, broiler birds stocked at medium or high density had significantly decreased expression levels of both genes in the liver tissues compared with those stocked at low stocking density. Comparing the MSD and HSD groups, the expression levels of GH and IGF-1 genes were significantly improved by dietary supplementation of Vit E or Zn, but they did not differ from the LSD group.

### Economic efficiency

Table 4 shows the effects of different SDs and the influence of Vit E or Zn supplementation to medium or high stocked birds on the economic parameters and economic efficiency measures during the experimental period. As for the costs of starter (1st and 2nd weeks) and grower (3rd and 4th weeks) diets, there were no significant differences among the LSD and other groups either supplemented or not supplemented with Vit E or Zn. However, the cost of the finisher diet showed a significant (*p* < 0.05) difference among the different groups. The MSDZn group showed higher finisher diet costs, followed by the HSDVE and MSDVE groups. The broiler sale, TR, and NP differed significantly in the LSD group compared with the other experimental groups.

**Table 4:** Effect of different stocking densities (SDs) and the influence of vitamin E (Vit E) or zinc (Zn) supplementation to medium or high density stocked birds on the economic parameters and economic efficiency measures during the experimental period of broiler chickens

Treatment (T)												
Items	SD			SD-treated Vit E		SD-treated Zn		Pooled SEM		*P*-value		
	LSD	MSD	HSD	MSDVE	HSDVE	MSDZn	HSDZn		T^1^	C vs SD	C vs SD + vit E	C vs SD + zinc
Economic indices (LE/bird)												
Chick price	5.50	5.50	5.50	5.50	5.50	5.50	5.50	0.00		–	–	–
Drug cost	1.30	1.30	1.30	1.30	1.30	1.30	1.30	0.00		–	–	–
Vaccine cost	0.25	0.25	0.25	0.25	0.25	0.25	0.25	0.00		–	–	–
Disinfectant cost	0.30	0.30	0.30	0.30	0.30	0.30	0.30	0.00		–	–	–
TVM	1.85	1.85	1.85	1.85	1.85	1.85	1.85	0.00		–	–	–
Water & Elec	0.15	0.15	0.15	0.15	0.1 5	0.15	0.15	0.00		–	–	–
Equipment	2.00	2.00	2.00	2.00	2.00	2.00	2.00	0.00		–	–	–
Labor	1.50	1.50	1.50	1.50	1.50	1.50	1.50	0.00		–	–	–
Litter cost	1.25	1.25	1.25	1.25	1.25	1.25	1.25	0.00		–	–	–
Building	2	2	2	2	2	2	2		–	–	–	
TFC	2.15	2.15	2.15	2.15	2.15	2.15	2.15	0.00		–	–	–
Starter cost	3.64	3.53	3.63	3.53	3.67	3.61	3.74	0.03	NS	0.94	0.98	0.99
Grower cost	8.41	8.48	8.87	9.29	8.83	9.44	9.13	0.10	NS	0.86	0.26	0.05
Finisher cost	10.23^b^	10.12^b^	10.31^b^	10.68^ab^	10.41^b^	11.17^a^	10.28^b^	0.06	*	1.00	0.33	0.06
Feed cost	22.28^b^	22.13^b^	22.81^ab^	23.50^ab^	22.91^ab^	24.22^a^	23.14^ab^	0.14	*	0.98	0.25	0.05
Litter return	0.55	0.55	0.55	0.55	0.55	0.55	0.55	0.00		–	–	–
Broiler sale	48.61^a^	42.67^c^	42.37^c^	47.42^ab^	44.79^bc^	49.32^a^	45.53^b^	0.18	**	< 0.01	< 0.01	0.21
TVC	32.53^b^	32.38^b^	33.06^ab^	33.75^ab^	33.16^ab^	34.47^a^	33.39^ab^	0.15	**	0.98	0.25	0.05
TC	34.68^b^	34.53^b^	35.21^ab^	35.90^ab^	35.31^ab^	36.62^a^	35.54^ab^	0.15	**	0.98	0.25	0.05
TR	49.16^a^	43.22^c^	42.92^c^	47.97^ab^	45.34^bc^	49.87^a^	46.08^b^	0.18	**	< 0.01	< 0.01	0.21
NR	14.48^a^	8.69^c^	7.70^d^	12.07^ab^	10.03^bc^	13.25^a^	10.54^b^	0.20	**	< 0.01	< 0.01	0.00
Economic efficiency measures												
TR/TVC	1.51^a^	1.34^ cd^	1.30^d^	1.42^b^	1.37^c^	1.45^ab^	1.38^bc^	0.01	**	< 0.01	< 0.01	< 0.01
NR/TC	0.42^a^	0.25^ cd^	0.22^d^	0.34^b^	0.28^c^	0.36^ab^	0.30^bc^	0.01	**	< 0.01	< 0.01	< 0.01
NR/TVC	0.45^a^	0.27^ cd^	0.23^d^	0.36^b^	0.30^c^	0.38^ab^	0.32^bc^	0.01	**	< 0.01	< 0.01	< 0.01
BW (kg)	2.31^a^	2.03^c^	2.02^ cd^	2.26^ab^	2.13^bc^	2.35^a^	2.17^b^	0.01	**	< 0.01	< 0.01	0.21
BWG (kg)	2.27^a^	1.98^c^	1.97^ cd^	2.21^ab^	2.08^bc^	2.30^a^	2.12^b^	0.01	**	< 0.01	< 0.01	0.21
BCR	1.42^a^	1.25^ cd^	1.22^d^	1.34^b^	1.28^c^	1.36^ab^	1.30^bc^	0.01	**	< 0.01	< 0.01	< 0.01

^1^Means within a row with different superscripts (a,b,c) significantly differ (*p < 0.05, **p < 0.01, and *NS*, not significnt). *SD*, stocking density; LSD, low stocked denisity; MSD, medium stocked density; *HSD*, high stocked density; *MSDVE*, medium stocked density birds supplemented with Vit E; *HSDVE*, high stocked density birds supplemented with Vit E; *MSDZn*, medium stocked density birds supplemented with Zn; *HSDZn*, high stocked density birds supplemented with Zn; *TVM*, total veterinary management; *TFC*, total feed cost; *TVC*, total variable cost; *TC*, total costs; *TR*, total return; *NR*, net return; kg, kilogram; *BCR*, benefit cost ratio; and *SEM*, standard error of the mean

Higher values were observed for broiler sales and TR in the MSDZn groups, followed by the LSD group. In contrast, lower broiler sales and TR were detected in the MSD and HSD groups. The highest values of NR were observed in the LSD, MSDZn, and MSDVE groups. Economic efficiency measures revealed that TR/TVC, NP/TC, NP/TVC, and BCR were significantly increased in the LSD group followed by the MSDZn, MSD, and HSD groups. At the same time, BW/kg and BWG/kg were found to considerably increase in the MSDZn group, followed by the LSD, MSD, and HSD groups.

## Discussion

In modern livestock production systems, the intensification of production is one of the measures applied to meet the increasing demand for animal protein sources. However, stocking animals at a high density is always related to increased health hazards, and it requires good husbandry practices by the farmers. Intensive farming increases the sensitivity of birds to external stressors from their surrounding environment resulting in acute stress responses, reduced productivity, health problems, and low-quality poultry products (Li et al. 2019), thus achieving the balance between intensive farming and animals’ health levels while keeping the production process profitable is problematic.

In this study, we investigated the effects of different SDs to find the density that does not negatively impact the health performance of birds, and we also aimed to find an effective and simple measure to mitigate the adverse effects of HSD. These investigations were underpinned by economic evaluation to achieve a practical and economic recommendation for the breeders who have to breed their birds intensively. This study showed that HSD (13 and 15 birds/m^2^) negatively impacted the overall BW and BWG. These results are in agreement with those previously reported in several studies in which the ideal SD of broiler birds was between 7 to 10 birds/m^2^ (Gasparinoa et al. 2014; Mohammed et al. 2021a). In fact, the decreases in FI by birds stocked at high densities can be one of the factors that negatively affect BW and BWG, specifically at grower and finisher phases. Increasing weights/sizes of the birds during fattening may hindrance access to feeders, increasing the competition between birds to get to the feeders (Tong et al. 2012).

Birds stocked at high densities showed homeostasis changes specifically at the finisher phase, indicating the presence of stresses. These birds had high blood plasma glucose, T_3_, T_4_, and corticosterone. Biological and/or production stressors such as SD, heat, cold, cooping, restraint, and shackling evoke corticosterone synthesis in birds (Dozier et al. 2006; Alagawany et al., 2018). Furthermore, Antar et al. (2020) reported that stressors can increase the circulating concentrations of T_3_ and T_4_. The increase in blood plasma glucose of birds stocked at high densities is a normal result of increased corticosterone concentrations. Increased glucocorticoid concentrations are combined with increased concentrations of circulating glucose in chickens (Wang et al. 2012). The increases in circulating concentrations of glucose are likely to be due to increased rates of hepatic gluconeogenesis stimulated by the action of glucocorticoids and corticosterone. Other reasons for the increases in circulating glucose concentrations are probably due to the reduced utilization of glucose (Scanes 2016). The decreased growth rate by corticosterone was associated with the energy expenditure, proteolysis, and gluconeogenesis, by vital tissues such as muscles (Lin et al. 2004). Furthermore, high concentrations of corticosterone can significantly reduce FI and thus BWG in broiler birds (Luo et al. 2013).

In this study, the expressions of hepatic GH and IGF-l genes were downregulated by overcrowding at a molecular level. This was in harmony with results obtained by Liu et al. (2019), who stated that high SD harmed muscle and bone growth with a lower m-RNA expression of IGF-l. In context, Beccavin et al. (2001) found a positive association between the BW of broiler birds and hepatic IGF-I m-RNA expression levels. In growing broiler birds, IGF-l induces growth of the skeletal muscles by enhancing the protein synthesis rate (Wen et al. 2014). Overall, these metabolic and molecular changes with low FI explain the significant reductions in growth performance attributes of birds stocked at high densities (13 or 15 birds/m^2^).

Economically, stocking birds at high densities results in huge economic loss and low profitability as indicated by the decreased TR, NR, and other economic efficiency attributes. In fact, these economic losses were mainly due to the sensible decreases in BW and BWG, but not feeding costs, as FI was low in the birds stocked at high densities. This elucidates the decreased TVC values observed in the groups of birds stocked at high densities as feeding costs in poultry farms represent about 70–80% of the TVC (Al-Sagheer et al. 2019).

As an attempt to mitigate the negative impacts of overcrowding in poultry farms, we used either Vit E or Zn as feed additives for birds stocked at high densities. Generally, dietary supplemental vitamins and minerals can either boost the birds’ growth rates or alleviate negative effects of environmental and biological stresses (Shah et al. 2018; Hashem et al. 2017). Vit E can be adopted to enhance the growth performance of different farm animals, including broiler chickens, particularly under harsh environmental conditions and other stresses. In addition, Zn supplementation to broilers’ feed has positive effects on the growth performance of broilers and could increase the average daily gain and the FCR. In fact, we suggest that the requirements of vitamins and minerals under unfavorable environmental conditions are increased as different stressors increase the depletion and elimination of these vital molecules and worsen the animals’ overall health status (Hashem et al. 2017; Bortoluzzi et al. 2020).

In this study, the addition of Vit E or Zn to broiler birds stocked at high densities improved their growth performance as indicated by higher BW, BWG, and FCR. These results are inconsistent with reports of previous studies where Vit E supplementation to the birds stocked at high densities had better growth performance than those that did not receive Vit E supplementation (Elkhatim et al. 2020). Similarly, EL-Gogary et al. (2020) found that adding Zn in the diet for broiler chickens has beneficial effects on broiler birds’ growth performance and economic efficiency. The positive effects of either Vit E or Zn on growth performance can be ascribed to the ability of both supplementations to improve most physiological events impaired by the action of HSD. Birds stocked at high densities and supplemented with either Vit E or Zn showed a pattern of blood glucose concentrations (decreased compared to non-supplemented groups stocked at high densities) similar to those in birds stocked at low density, indicating the lack of negative effects of HSD on glucose metabolism. In fact, the decreases in blood plasma glucose concentrations in supplemented groups and LSD match the decreases in blood plasma corticosterone, as a reverse relationship is known between glucose concentrations and corticosterone. These decreases in blood plasma glucose could presumably result from insulin action that plays an essential role in regulating carbohydrates, glucose uptake, and lipid metabolism and stimulating growth by increasing protein synthesis and affecting the expression of growth-related genes (Taniguchi et al. 2006). However, our finding disagreed with the report that SD did not affect glucose concentrations (Abudabos et al. 2013).

This study observed significant decreases in stress-related hormones, including T3, T4, and corticosterone, in the LSD, MSDZn, HSDZn, MSDVE, and HSDVE groups. Several studies have shown that stress increased circulating concentrations of T_3_, T_4_, and corticosterone (Tareen et al. 2017). Actually, these hormones are pivotal for bone and muscle growth in broilers but only under physiological conditions (Li et al. 2019). In our study, Vit E or Zn supplementation upregulated GH, and IGF-1 m-RNA expression restored the negative effect of overcrowding on the broiler chicken’s growth. This is in line with the findings obtained by Hosseini-Mansoub et al. (2011), who stated that higher BW is obtained when birds received Vit E (100 mg/kg diet) or Zn (50 mg/kg diet) compared with control birds. Furthermore, Kirrella et al. (2021) suggested that the relative m-RNA expression of IGF-I and GH receptor genes was upregulated with the addition of corn silk meal in the broiler diet because of its higher Vit E content and other elements like Zn minerals. Additionally, Zn supplementation to male broilers elevated the serum concentrations of IGF-I (Tomaszewska et al. 2017). Also, Zn is required for the normal activity of numerous enzymes, structural proteins, and hormones, and is essential for growth and development, regulation of gene expression, and protein synthesis (Lin et al. 2004).

From an economic point of view, supplementing Zn to MSD birds (MSDZN) resulted in TR, NR, and other economic efficiency comparable to those recorded for MSD and HSD birds. This effect is mainly due to the improvements in all growth performance-related variables, which may compensate for the high costs of Vit E- or Zn-supplemented diets. There is a direct relationship between broiler sales and final BW, reflecting TR and NP (Tomaszewska et al. 2017; Kirrella et al. 2021).

## Conclusion

Our results indicate that stocking broiler birds at a level of 10 birds/m^2^ are considered an ideal stocking density for Cobb-500 broilers. At this SD, birds can express adequate growth performance with no signs of stress and provide sufficient economic profit. However, if a breeder intends to apply an intensive farming system with the same production outputs, it is recommended that the SD should be 15 birds/m^2^ with Zn supplementation at a level of 100 mg/kg DM diet during the whole fattening cycle. This is an efficient measure to maintain the birds’ welfare and to achieve adequate growth performance and economic profit.

## Data Availability

Data are available upon request.

## Abbreviations

*SD*: Stocking density
*LSD*: Low stocked denisity
*MSD*: Medium stocked density
*HSD*: High stocked density
*MSDVE*: Medium stocked density birds supplemented with Vit E
*HSDVE*: High stocked density birds supplemented with Vit E
*MSDZn*: Medium stocked density birds supplemented with Zn
*HSDZn*: High stocked density birds supplemented with Zn
*IBW*: Initial body weight
*FBW*: Final body weight
*BWG*: Body weight gain
*FI*: Feed intake
*FCR*: Feed conversion rate
*TVM*: Total veterinary management
*TFC*: Total feed cost
*TVC*: Total variable cost
*TC*: Total costs
*TR*: Total return
*NR*: Net return
*kg*: Kilogram
*BCR*: Benefit cost ratio
*GH*: Growth hormone
*GHR*: Growth hormone receptor
*HSD*: High stocking density
*IGF1*: Insulin-like growth factor 1
*RNA*: Ribonucleic acid
*IGF-l*: Insulin-like growth factor-l
*T*_*3*_: Triiodothyronine
*T*_*4*_: Thyroxin
*cortico*: Corticosterone
